# Urban-rural inequalities in suicide among elderly people in China: a systematic review and meta-analysis

**DOI:** 10.1186/s12939-018-0881-2

**Published:** 2019-01-03

**Authors:** Meizhi Li, Srinivasa Vittal Katikireddi

**Affiliations:** 1 Department of Public Health, University of Glasgow, Glasgow, UK; 2 The Second Xiangya Hospital of Central South University, Changsha, China; 3 MRC/CSO Social & Public Health Sciences Unit, University of Glasgow, Glasgow, UK

**Keywords:** Suicide, China, Aged, Elderly, Urban, Rural, Disparities, Inequalities, Geographic patterns

## Abstract

**Background:**

China has an unusual pattern of suicides, with overall suicide rates in rural areas higher than urban areas. While suicide rates have decreased dramatically, older people increasingly contribute to the overall burden of suicide. However, it is unclear if elderly people within rural areas experience greater suicide risk than those in urban areas. We aimed to systematically review the incidence of suicide in rural and urban China among the elderly (aged over 60 years), with a view to describing the difference in rates between rural and urban areas and trends over time.

**Methods:**

Chinese and English language articles were searched for using four databases: EMBASE (Ovid), MEDLINE (Ovid), PsycINFO (EBSCOhost) and CNKI (in Chinese). Articles describing completed suicide among elderly people in both rural and urban areas in mainland China were included. The adapted Newcastle-Ottawa Scale (NOS) was used to assess risk of bias. One reviewer (ML) assessed eligibility, performed data extraction and assessed risk of bias, with areas of uncertainty discussed with the second reviewer (SVK). Random effects meta-analysis was conducted. Suicide methods in different areas were narratively summarised.

**Results:**

Out of a total 3065 hits, 24 articles were included and seven contributed data to meta-analysis. The sample size of included studies ranged from 895 to 323.8 million. The suicide rate in the general population of China has decreased in recent decades over previous urban and rural areas. Suicide rates amongst the elderly in rural areas are higher than those in urban areas (OR = 3.35; 95% CI of 2.48 to 4.51; I^2^ = 99.6%), but the latter have increased in recent years. Insecticide poisoning and hanging are the most common suicide methods in rural and urban areas respectively. Suicide rates for these two methods increase with age, being especially high in elderly people.

**Conclusions:**

The pattern of suicide in China has changed in recent years following urbanisation and aging. Differences in suicide rates amongst the elderly exist between rural and urban areas. Addressing the high suicide rate amongst the elderly in rural China requires a policy response, such as considering measures to restrict access to poisons.

**Electronic supplementary material:**

The online version of this article (10.1186/s12939-018-0881-2) contains supplementary material, which is available to authorized users.

## Background

Suicide is a substantial public health issue worldwide [1, 2] and has attracted the attention of researchers for a long time [3, 4]. However, unlike other countries that have the complete data of suicide rates since the early 1950s, the figures for suicide in China were almost entirely unavailable before the 1990s [5, 6]. After the release of data on suicide rates, China was reported as one of the countries with high suicide rates from the 1990s to the 2000s [7], but the suicide rate in China declined rapidly from 15.4 per 100,000 in 2000 to an average of 9.8 per 100,000 between 2009 and 2011 [8]. Moreover, the pattern of suicides previously in China was quite different when compared with other parts of the world. This was mainly reflected in two ways: first, a greater frequency of completed suicides among females than males; and second, rural areas exhibited a rate three times higher than that of urban populations [9, 10].

The evidence from previous research suggests that suicide rates are responsive to social changes such as aging and urbanisation [11]. China has the largest elderly population in the world [12]; the World Health Organization (WHO) estimate that the elderly population in China will potentially increase to 330 million by 2050, with China having over 30% of the population aged 60 years and over [13]. Given the very high elderly suicide rates compared to other age-groups and the unprecedented population aging in China, research on this topic will continue to be a major policy priority for the future. Thus, an understanding of elderly suicides and their determinants is urgently needed to design responsive prevention policies. In terms of rural-urban differences in suicide rates, contrary to the common belief that urbanisation is harmful for mental health and increases suicide rates [14], rural people in China have higher crude suicide rates [15]. From 2009 to 2011, 79% of all suicides occurred among rural residents [16]. Thus, the suicide rate in rural areas in China requires urgent focus as part of suicide prevention in China. While suicide rates are higher amongst the elderly and in rural areas, it remains unclear if differences exist between suicide rates amongst elderly people living in urban and rural areas. Furthermore, trends over time and the most common suicide methods are unknown.

Analyses of data about suicide can inform suicide prevention efforts. Understanding how the distribution of suicide methods can also contribute to suicide rates, as this can inform suicide prevention programmes or initiatives and target those methods in different areas that are more amenable to prevention. The suicide prevention strategy in China mainly includes restricting the access of suicide method and psychological intervention [5, 7]. Compare to the group of adolescent and school-age children, elderly suicide attracts less attention and there are currently no practical prevention strategies targeting to elderly people. In order to maintain the decreasing trend in suicide rate in China, tailored prevention measures are necessary to address the unique changing pattern of suicide risk among differing subgroups. We therefore aimed to systematically describe inequalities in suicide rates between urban and rural China, among elderly people.

## Methods

We developed a pre-defined protocol for this systematic review and registered it on PROSPERO. But due to delays in processing, a registration number was not issued prior to the review’s completion. A protocol is included in Additional file 1.

### Search strategy

A preliminary literature search was conducted to identify relevant search terms. Four main databases, EMBASE (Ovid), MEDLINE (Ovid), PsycINFO (EBSCOhost), and CNKI (China National Knowledge Infrastructure, in Chinese), were used to identify relevant studies. Search terms included ‘elder’, ‘aged’, ‘geriatrics’, ‘China’ and ‘suicide’ were used to find related articles. The search strategy included an electronic and manual search, developed in collaboration with medical librarians. The electronic literature search ended on 8th May 2017. The full search strategy used is contained in the Additional file 2.

The reference lists of included articles and citing lists of included articles (using Google Scholar) were searched to find other potential relevant articles (forward and backward citation searching). The reference lists of relevant systematic reviews were also searched for any unidentified articles. Endnote X7 was used to manage the reference lists.

### Inclusion and exclusion criteria

Articles which involved participants 60 years and over (the official definition of elderly in China [17]) and which reported rural-urban differences in suicide were included. Included articles had to present data for both rural and urban areas. This review considered articles that were conducted in mainland China, with articles conducted in Hong Kong, Macau and Taiwan excluded. The primary outcome of this study relates to people who completed suicide and the secondary outcome was the suicide methods used. Only completed suicides have included in this systematic review since attempted suicide may be subject to greater ascertainment bias, with case detection influenced by access and use of healthcare. Articles which considered differences between urban and rural areas in elderly suicide methods were included. There was no restriction imposed for type of study, language, year of publication or region of publication.

### Screening and selection of articles

After duplicate results were removed, screening was carried out on the basis of title, or title and abstract, by one reviewer (ML). Full texts of all potentially relevant articles were retrieved, with a low threshold for keeping the article in until the full text stage maintained. After obtaining the full-text articles, one reviewer (ML) then determined whether these articles met the inclusion criteria. In the case of uncertainty, articles were discussed with the second reviewer (SVK). Reasons for excluding full text articles were noted for all full text articles (Additional file 3).

### Data extraction

A standardised data extraction template was developed using Microsoft Excel 2016 to extract characteristics and data from each article. A detailed template was created to extract relevant information (Additional file 4). For included articles, data about suicide rates and time periods for which they applied among both rural and urban elderly people were extracted and processed on Microsoft Excel 2017 to draw the graph of trends over time. When rates applied to multiple years, the midpoint was used for the purpose of illustration. All suicide data extracted for meta-analysis were converted to Odds Ratios (ORs) using standard approaches. In order to calculate the ORs, where the numbers of suicides or non-suicides were known or could potentially be calculated, a 2 × 2 table was created from these and used in meta-analysis.

The odds ratio (OR), standard error (SE) and 95% confidence interval (95%CI) were calculated by using these formula [18]:

$$ OR=\frac{\mathrm{a}/\mathrm{b}}{\mathrm{c}/\mathrm{d}}=\frac{\mathrm{a}\times \mathrm{d}}{\mathrm{b}\times \mathrm{d}} $$

$$ \mathrm{SE}=\mathrm{square}\ \mathrm{root}\ \mathrm{of}\ \left(1/\mathrm{a}+1/\mathrm{b}+1/\mathrm{c}+1/\mathrm{d}\right) $$

$$ 95\%\mathrm{CI}=\exp .\left(\ln \left(\mathrm{OR}\right)-1.96\times \mathrm{SE}\ \left[\ln \left(\mathrm{OR}\right)\right]\right)\ \mathrm{to}\ \exp .\left(\ln \left(\mathrm{OR}\right)+1.96\times \mathrm{SE}\ \left[\ln \left(\mathrm{OR}\right)\right]\right) $$

### Critical appraisal and data synthesis

Critical appraisal was done to assess risk of bias (sometimes referred to as quality) of included articles. An adapted version of the Newcastle Ottawa Scale (NOS) [19] was used to score individual articles (Additional file 5). The NOS has eight items with the highest possible score nine and the lowest possible score zero. Articles with a score of 7–9 were deemed to have low risk of bias, 5–6 moderate risk of bias and those with a score of 1–4 high risk of bias in this review.

Both narrative synthesis and meta-analysis were used to analyse the data from included articles, with risk of bias considered throughout the synthesis process [20]. Narrative synthesis was conducted to describe differences in suicide rates between rural and urban areas, trends in suicide rates over time, differences in suicide methods between rural and urban areas. Tables and figures were used to describe the data in the preliminary synthesis. Detailed templates which present a summary of descriptive data from all of the included articles were then created. Our synthesis focused on addressing three issues: first, establishing whether differences in suicide rates between rural and urban areas existed amongst the elderly; second, to describe trends over time; and third, to describe the methods used for completed suicide. Tabulation was primarily intended to facilitate comparisons across urban and rural areas in relation to the method of suicide, as well as distinguishing between articles at high and low risk of bias. We stratified analyses by gender and age group, as allowed by the data reported. To make comparisons in inequalities over time, the ratio of suicides among rural to urban areas was also calculated.

Meta-analysis was used to calculate a summary estimate of effect size for the difference between suicide rates in urban and rural areas (with the urban area as the reference group). Following this, we explored reasons for differences in effects between and among articles, and to identify heterogeneity in the effects of the intervention in different subgroups [21]. Heterogeneity was explored by pre-specified sub-group analyses, according to the category of ages, the study period, the level of region (multiple or individual areas) and the final score in each included article after critical appraisal. We planned to look for evidence of publication bias using a funnel plot if ten or more articles were available for meta-analysis. Ethical approval was not sought for this study.

## Results

**Fig. 1:**
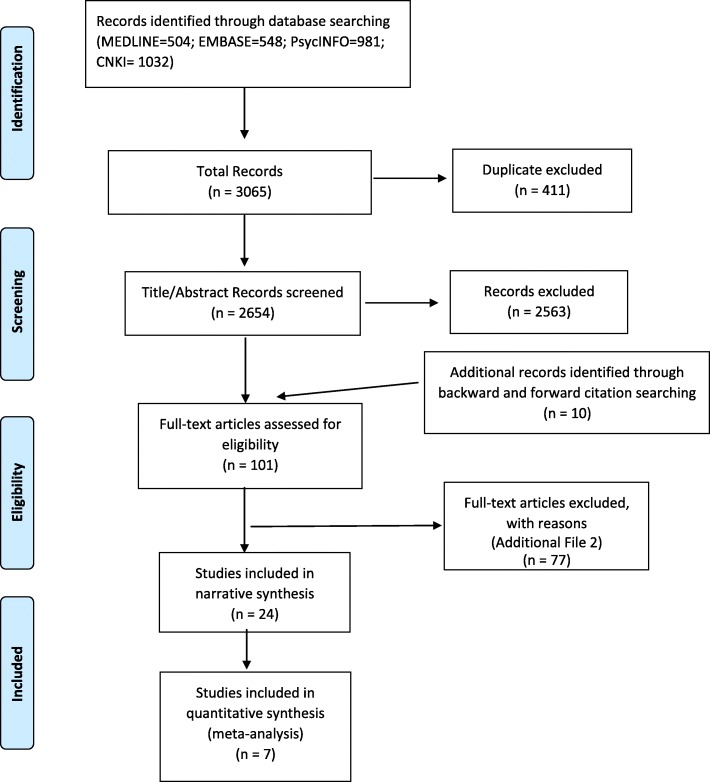
PRISMA flow diagram of study selection

The electronic database search returned 3065 results, with 2654 hits left after duplicates were eliminated (see Fig. 1). Ninety-one full text articles were retrieved from these and manual searches yielded a further ten potentially relevant articles. In total, 24 articles met the inclusion criteria for the narrative synthesis, with seven of these articles providing numerical data suitable for meta-analysis.

### Narrative synthesis of articles

#### Characteristics of included articles

The main characteristics of the included articles are summarised in Table 1. The smallest sample size in the included articles was 895 [22] whilst the largest was around 323,800,000 (about 25% of total population in China) [23]. All included articles contained both females and males. The included articles contained provinces (the highest level in Chinese administrative divisions [24]), cities and counties in China: Yunnan province [25], Shandong province [26], Hunan province [27], Beijing city [28, 29], Chengdu city [30], Changsha city [27], Zhuhai city [31], Yantai city [32], Liuyang county [27] and Laiyang County [32]. All of the 24 included articles analysed completed suicides in both urban and rural areas. Thirteen [22, 25–28, 30–37] of these articles provided information about suicide methods. The year of publication of the included articles covered the time period from 1993 to 2017. The study period of data collection for included articles was mostly from 1987 to 2014, but one article [33] analysed data from 1968.

**Table 1:** Characteristics of included articles

Author	Year of publication	Study period	Region level	Risk of bias	Study design	Population size	Source of data	Suicide methods
He, et al. [33]	1998	1968–1992	Multiple regions	High	Review	Not stated	“Appeared in Chinese publications” (Not clear)	Yes
Ji, et al. [34]	2001	1988, 1990, 1992	Multiple regions	Low	Review and Secondary data analysis	Not stated	The Chinese Ministry of Public Health (CMPH)	Yes
Li, et al. [35]	2009	Not Applicable	Multiple regions	High	Review	Not stated	Not mentioned	Yes
Lu, et al. [25]	2013	2004–2005	Yunnan Province	Moderate	Register studies	8,128,780	The Ministry of Health Vital Registration (MOH-VR) system, Police departments, Civil Affairs departments, Medical Institutions and Family Planning Departments	Yes
Page, et al. [36]	2017	2006–2013	Multiple regions	Low	Register studies	73,000,000	The Chinese Disease Surveillance Points (DSP) system	Yes
Phillips, et al. [37]	2002	1995–1999	Multiple regions	Low	Register studies	110,000,000	The Chinese Ministry of Health (MOH)	Yes
Sha, et al. [43]	2017	1990–2010	Multiple regions	Moderate	Register studies	Roughly 8% of the national population	The Chinese Ministry of Health Vital Registration (MOH-VR) System	No
Sun, et al. [26]	2013	1991–2010	Shandong province	Moderate	Register studies	12,000,000 (13% of the total population in the province)	Shandong Disease Surveillance Point (DSP)	Yes
Sun, et al. [41]	2014	2002–2011	Multiple regions	Low	Register studies	8% of the national population	The Ministry of Health (MOH) of the People’s Republic of China	No
Wang, et al. [16]	2014	2002–2011	Multiple regions	Low	Register studies	8% of the national population	The China’s Ministry of Health Vital Registration (MOH-VR) system	No
Yang, et al. [22]	2005	1996–2000	Multiple regions	Moderate	Cross-sectional studies	895	23 of the 145 National Disease Surveillance Point (NDSP) system	Yes
Yip, et al. [28]	2000	1991–1996	Beijing city	Moderate	Register studies	Not stated	Beijing Public Security Bureau	Yes
Yip, et al. [44]	2005	1991–2000	Multiple regions	Moderate	Register studies	10% of the national population	The Ministry of Health (MOH) of the People’s Republic of China	No
Yip, et al. [7]	2008	1990–2000	Multiple regions	Low	Register studies	10% of the national population	The Ministry of Public Health (MOH) of the People’s Republic of China	No
Yip. [29]	2001	1987–1996	Beijing city	Low	Register studies	Not stated	The Ministry of Public Health (MOH) of the People’s Republic of China	No
Zhong, et al. (a) [23]	2016	1987–2014	Multiple regions	Low	Register studies	323,800,000	The Ministry of Public Health Vital Registration (MOH-VR) system of the People’s Republic of China and the Integrated National Mortality Surveillance System (INMSS)	No
Zhong, et al. (b) [39]	2016	2013–2014	Multiple regions	Low	Register studies	323,800,000	The Integrated National Mortality Surveillance System (INMSS)	No
Peng, et al. (In Chinese) [30]	2013	2006–2010	Chengdu city	Moderate	Register studies	15,436,893	Chengdu Death Registration Reporting Information system	Yes
Xu, et al. (in Chinese) [32]	1993	1988–1990	Yantai city and Laiyang rural area	Moderate	Case-control	878,951 (rural)/317,328 (urban)	Household death registration	Yes
Xu, et al. (in Chinese) [27]	2000	1990–1998	Hunan province: Changsha city and Liuyang county	Low	Register studies	Not stated	Hunan public health department	Yes
Li, et al. (in Chinese) [58]	2007	2004–2005	Zhuhai city	Moderate	Register studies	Not stated	Zhuhai Disease Surveillance system	Yes
Cai, et al. (in Chinese) [40]	2012	2010	Multiple regions	Low	Register studies	730,000,000	National Disease Surveillance (NDS) system	No
Wang (in Chinese) [42]	2013	2004; 2010	Multiple regions	Low	Register studies	Not stated	Chinese Statistical Year Book	No
Yan. (in Chinese) [38]	2003	1990; 1995; 2000	National (41 urban cities and 101 rural areas)	Low	Register studies	Not stated	Chinese Statistical Year Book	No

## Results of critical appraisal

Detailed scores of the critical appraisal are listed in Additional file 4. Fourteen articles were classified as low risk of bias, nine as moderate and one as high. Three articles [16, 23, 38] had the highest score of nine, six articles [27, 29, 36, 37, 39, 40] had a score of eight, four articles [34, 41, 42] had score of seven, six [22, 26, 28, 30, 43, 44] articles scored six, three articles [25, 31, 32] scored five, and two included articles have the score of four and three, separately [33, 35].

### Differences in suicide rates between urban and rural areas

Recent articles have found a big difference in suicide rates between rural and urban areas, especially among older adults [10, 45, 46]. Li et al. [35] highlighted that suicide in China showed unique demographic patterns with age: the highest completed suicide rate among the population group was over 65 and It is approximately four to five times higher than the rate in the general population. Overall, suicide rates among the older Chinese have fallen in both urban and rural areas, but more noticeable in rural areas [16, 35]. Moreover, few of the articles focused on elderly suicide rates with rural-urban differences.

For most of the recent years studied, for example, during the year from 2002 to 2011, the overall rural/urban ratios of elderly suicide rates varied between 1.5 and 2.5 [41], rural areas have continually had higher suicide rates than urban areas. All except one large study (at moderate risk of bias [25]) found increased risk of completed suicide amongst the elderly in rural areas. The magnitude of differences between urban and rural areas differed markedly. In the article of Li et al. [35], for groups aged 60 years and above, the number of completed suicides in rural areas was five to seven times higher than in urban areas, while the rural/urban ratios were less than 3:1 for all age groups.

**Fig. 2:**
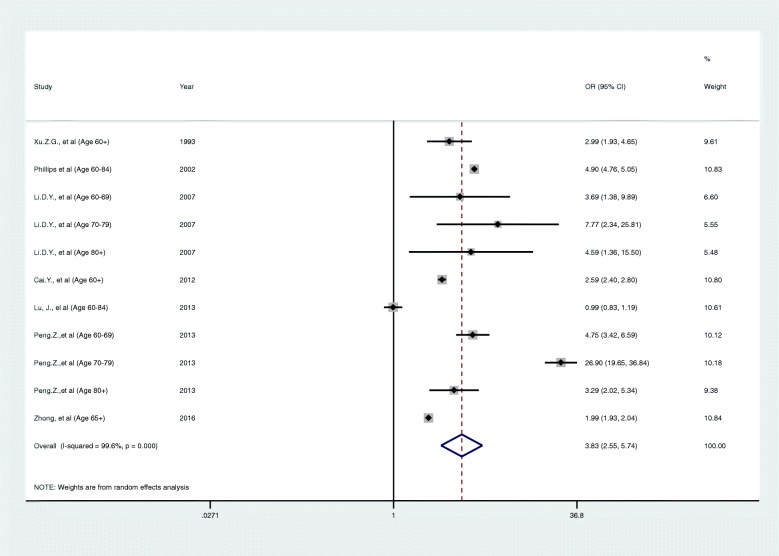
A forest plot of completed suicide amongst elderly people in rural areas compared to urban areas

Figure 2 shows the results of the meta-analysis pooling the odds ratios for completed suicides for rural areas compared to urban areas amongst the elderly. Suicides amongst the elderly occurred more frequently in rural areas (OR 3.35, 95% CI 2.48-4.51), although heterogeneity was high (I^2^ = 99.6%). Detailed data on the rates of suicide for Meta-analysis from included articles are included in Additional file 6.

**Fig. 3:**
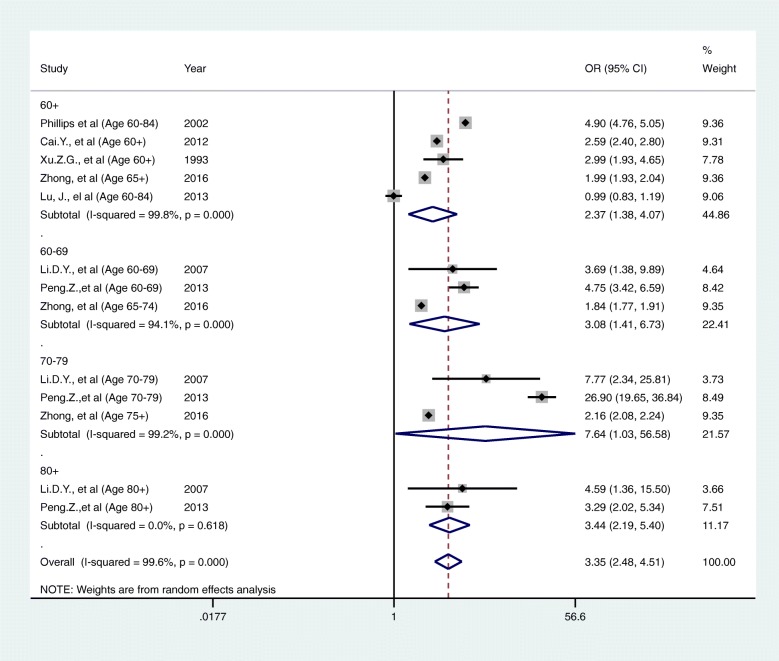
A forest plot of sub-group analysis according to category of ages

**Fig. 4:**
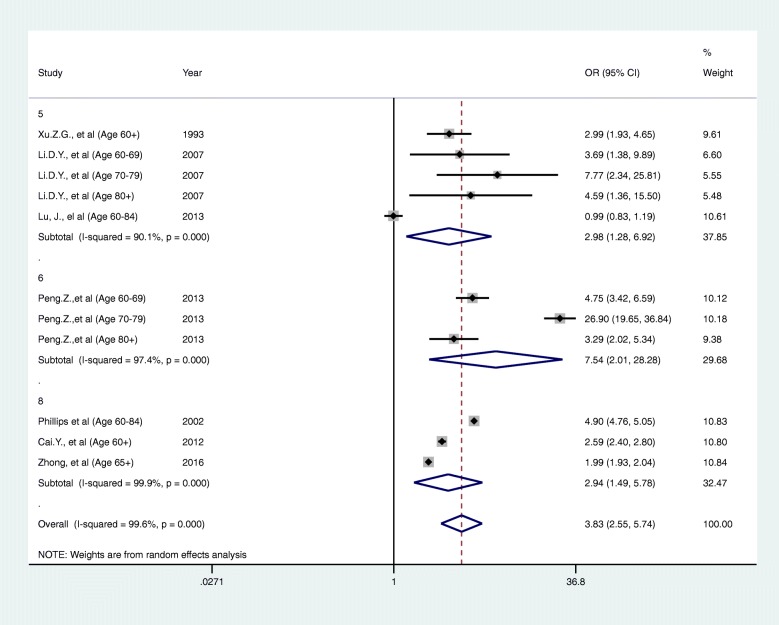
A forest plot of sub-group analysis according to category of score after critical appraisal

**Fig. 5:**
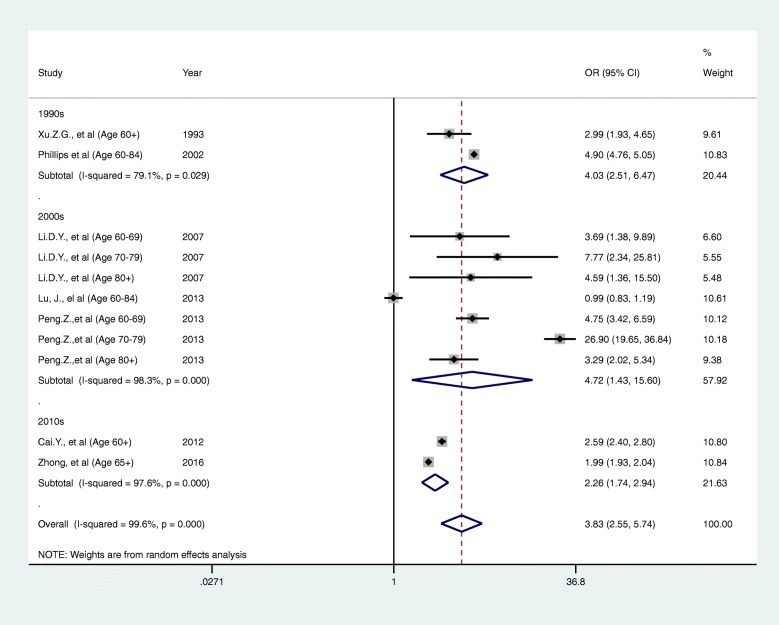
A forest plot of sub-group analysis according to category of study period

**Fig. 6:**
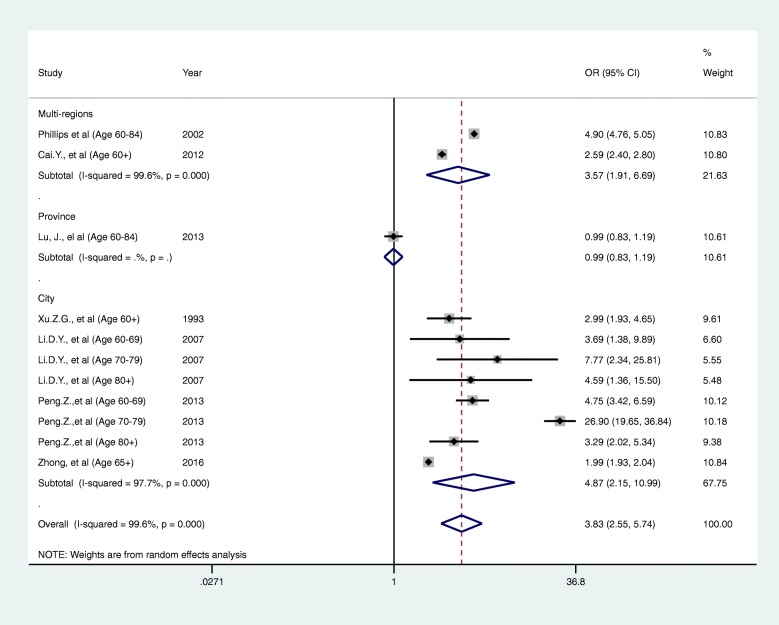
A forest plot of sub-group analysis according to category of region level

Given the high heterogeneity, we carried out subgroup analyses by age group, study period, geographical unit (multiple regions or individual areas) and risk of bias assessment were explored to investigate whether these factors explain the observed heterogeneity. We identified no clear explanatory factors from these analyses (Figs. 3, 4, 5 and 6). This could possibly because of a lack of articles available for subgroup analysis. Under these circumstances, the reason for the heterogeneity is unknown for now.

### Methods of suicide

Twelve out of the 24 articles described suicide methods used in rural and urban areas in China [22, 25–28, 30–37]. In these articles, commonly mentioned methods of suicide included poisoning, jumping, drowning, hanging, and medication overdose. Other methods such as fasting, poisoning by gas, self-burning, firearms and Seppuku (a form of Japanese ritual suicide by disembowelment [47]) were detected as occurring only in a few included articles [22, 28, 33]. Yip et al. [28] concluded that gas and firearms were used exclusively in the urban areas.

Only one article [27] of the included articles compared suicide methods amongst elderly people in both rural and urban areas in China. In the findings from this article, Xu el al [27] illustrated that the top three suicide methods among the elderly in urban areas were poisoning (29.4%), hanging (28.4%) and jumping (16.1%). As in rural areas more generally, poisoning (68.2%) accounted for the majority of suicide methods among elderly people, followed by hanging (23.7%) and drowning (5.8%). One article [35] compared the suicide methods of elderly people with general population. Li et al. [35] concluded that the ingesting of pesticides or rat poisons are common methods of suicide for both older age group and general population age groups.

The remaining 10 articles mainly explored the suicide methods between rural and urban China, without detailing age groups. In those articles, poisoning remained the commonest suicide methods in China, over time. Similar results in different articles illustrated that the most common method of suicide was hanging in urban areas, but insecticide poisoning in rural areas [22, 25, 31]. When comparing the results from articles with different geographical levels, similar findings were found. For example, in the multiple-regions level article of He et al. [33], it was suggested that the use of poisons was more common in rural areas and jumping less common, and this could also be found in the results at province-level article of Lu et al. [25]. Sun et al. [26] illustrated the trends in suicide methods between rural and urban areas during the period from 1991 to 2000: In urban areas, suicide rates due to pesticide were increasing but hanging continued to be the leading method. In rural areas, pesticide poisoning declined over time but it remained the top method.

### Trends over time

**Fig. 7:**
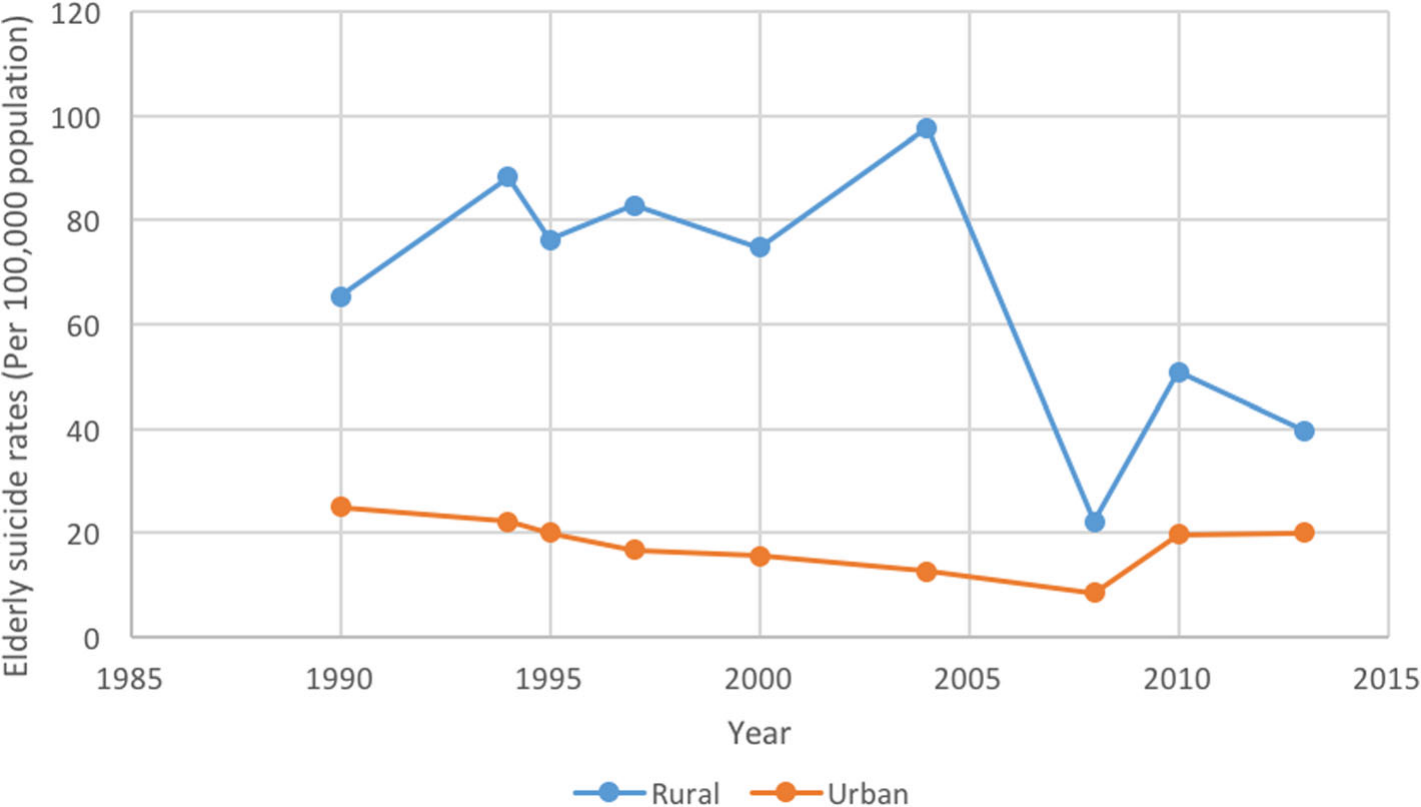
Suicide rates among elderly people over time in rural and urban China

Data were extracted from included articles to draw graphs showing trends over time in elderly suicide rates in China (Fig. 7). Differences between the overall suicide rates in both rural and urban areas have decreased in recent decades [34, 37], with elderly suicide rates in both urban and rural areas having decreased as well. Yip et al. [28] noted that elderly suicide rates in Beijing were six times higher than the general population; elderly suicide rates were consistently much higher than overall suicide rates over time, in both rural and urban areas.

**Fig. 8:**
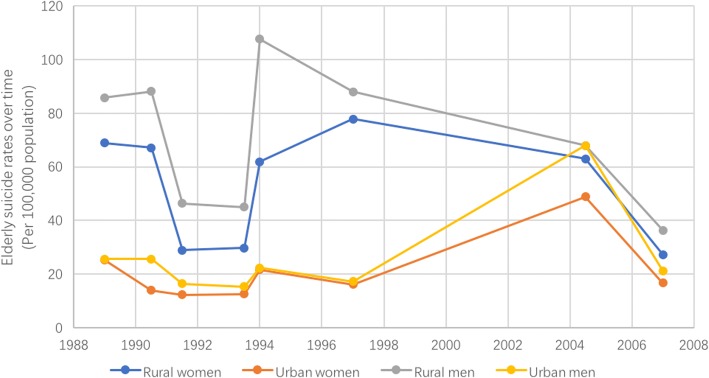
Suicide rates among elderly people over time in rural and urban China, stratified by gender

Figure 8 shows the gender-specific suicide rates for rural and urban elderly people in China. From 1991 to 1993, there was a decrease in suicide rates in both genders in rural and urban areas. However, rates started to increase again from 1993. Generally, the elderly suicide rates in rural areas were higher than rates in urban areas in both women and men. For both genders, the gap between rural and urban areas was getting smaller over this period of time. Moreover, in contrast to the previous pattern in China, it can be seen that suicide rates in elderly men are generally higher than the rates in women, over the period of time illustrated in the figure.

**Fig. 9:**
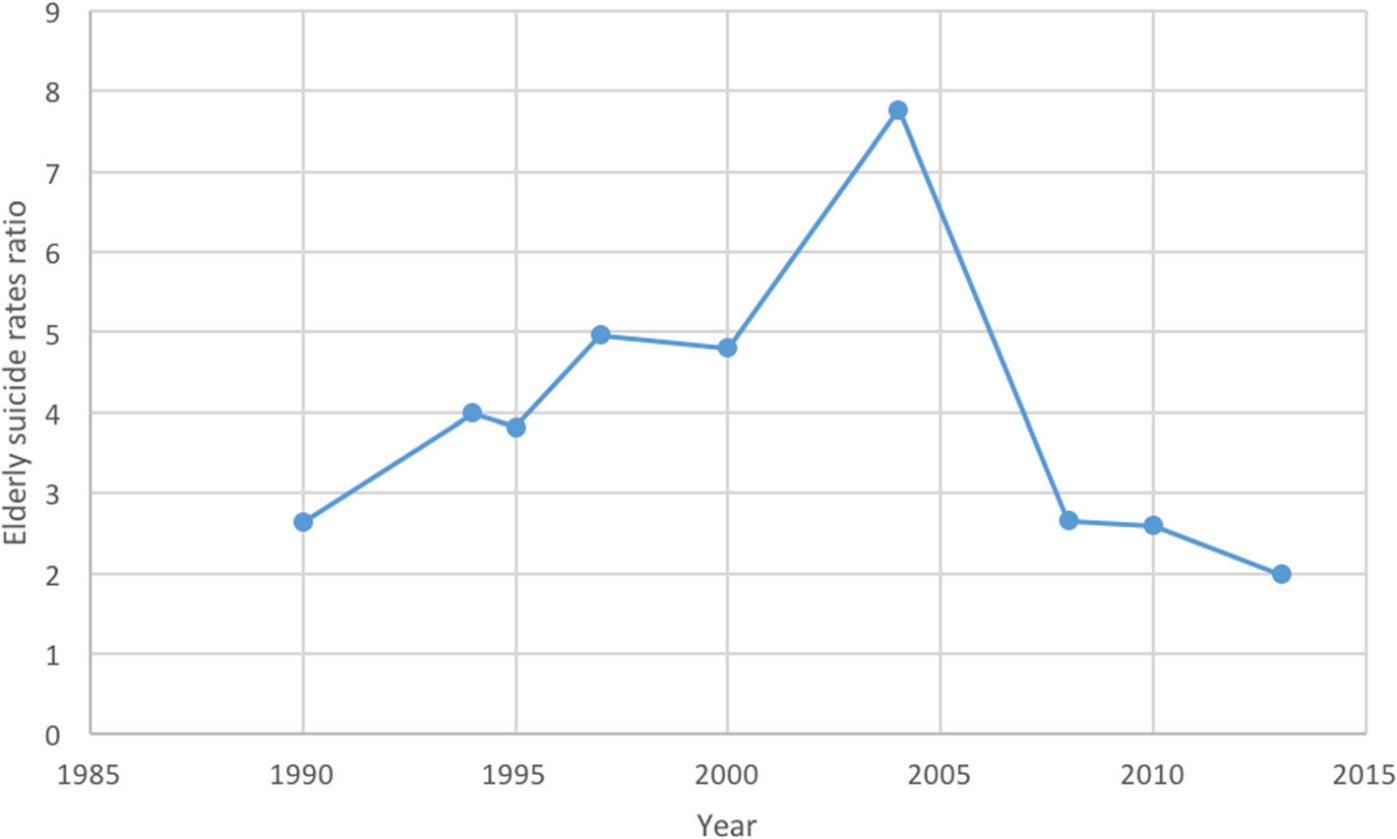
The ratio of elderly suicide rates in rural areas compared to urban areas over time

Figure 9 shows the trend over time in the ratio of rural/urban elderly suicide rates. It shows that the ratios over time have remained bigger than one, which means the elderly suicide rates in rural areas were consistently higher than urban areas over time. The ratio increased since the early 1990s until 2004 but has subsequently fallen since 2004.

## Discussion

### Main findings

Inequalities in suicide rates between rural and urban areas have existed in China for a long time [34, 48]. This study has focused on understanding this regional disparity among elderly people. Our meta-analysis of data from seven articles [25, 30–32, 37, 39, 40] found substantially higher suicide rates amongst the elderly in rural areas (OR 3.35, 95%CI 2.48 to 4.51). There was only one article conducted in Yunnan [25] which found little difference in elderly suicide rates between rural and urban areas (OR 0.99; 95%CI 0.83 to 1.19). We identified very high heterogeneity (I^2^ = 99.6%) which remained unexplained, despite subgroup analyses. This might suggest that a different pattern of elderly suicide rates exists between individual regions of China, but further investigation of this heterogeneity is required in future research. Previous articles reported that the suicide rates in rural areas were more than three times higher than the rate in urban areas [44]. The rural/urban suicide rates ratio is now less than 2.0, which indicates that while a substantial decrease has occurred in China [10], regional inequality still exists. The narrative synthesis has found consistently higher rates of suicide amongst the elderly in rural areas, with these inequalities having increased from the 1990s but subsequently starting to decline. The underlying mechanisms for these fluctuations are unclear, but may be a result of the changes in China’s social and political status, for example, improved economic circumstances, improved life and more independence for rural young women in recent years [16]. With regards to methods of suicide, different methods were used in rural and urban areas in China. Poisoning is still the most frequently used method of suicide among rural residents in China while hanging continued to be the leading suicide method in urban areas [26, 36]. However, there was only one paper [35] which studied suicide methods among elderly people in rural and urban areas identified in this review.

### Comparison with previous articles

The main findings from this systematic review are similar with previous articles but do have slight differences. Similar to the findings from the general population, a regional disparity in elderly suicide rates between rural and urban areas still exists and there is a decreasing trend in elderly suicide rates in recent times. However, the downward trend is more noticeable in rural areas, suggesting this has greatly contributed to the decrease in overall elderly suicide rates.

In the systematic review conducted by Simon el al [49], a similar search strategy with this project was used to find articles related to regional differences in elderly suicide rates across regions and countries. However, in that systematic review, suicidal ideation attempts and completed suicide rates were studied. The regional distribution in Simon et al’s article is between China, Hong Kong, Taiwan, Asian societies and Western countries while in our study we have focused differences between rural and urban areas in China. This systematic review, therefore presents a clearer picture of suicides among elderly in mainland China.

One previous article published in 2011 has explored suicide rates and trends over time in China. In this article, Zhang et al. [50] analysed general suicide rates and compared trends among rural-urban and gender groups. They concluded suicide rates were lower in urban areas and the downward trend in rural areas is faster than urban areas. However, no specific age group comparisons were studied. Our study focuses on a more specific age group analysis but without exploring the underlying reasons or risk factors underpinning the suicide pattern. Understanding the risk factors for suicide is an important next step to build on our work. Zhang and colleagues’ article [50] is a good example of how to conduct more detailed analysis of the unique suicide rates in China. Their article evaluated the potential role of economic development and population mobility in China as a determinant of change in suicide rates, but similar efforts remain to be conducted with a focus on the elderly.

Only one article conducted by Li et al. [35] illustrated the difference in elderly suicide methods between rural and urban areas. Furthermore, there has been no previous systematic review which explores the both the suicide rate and suicide methods specifically among elderly people in rural and urban areas in China.

### Strengths of this study

This study utilised clear and pre-defined eligibility criteria which were presented in a registered protocol. A comprehensive search strategy was developed, including forward and backward citation searching, in collaboration with a medical librarian. Both narrative synthesis and meta-analysis were used to provide a transparent estimate of a pooled effect size, as well as allowing a more detailed understanding of the epidemiology realized by narrative synthesis [51]. Focused on elderly people in this review has allowed more detailed exploration of suicide rates and methods. Furthermore, articles were limited to those from mainland China as previous articles have suggested that the pattern in suicide rates varied substantially across the world [52]. This systematic review therefore holds particular policy relevance for suicide prevention in China.

### Limitations of this study

The most major limitation of our review was the use of only one reviewer for many aspects of the study conduct. Unfortunately, this was necessary due to the unfunded nature of the study and for language reasons. Thus, potential bias may have arisen from the selection process of included articles and their critical appraisal. To mitigate this, a second reviewer was involved in discussing areas of uncertainty and maintained a low threshold for retaining articles in the study selection process, until the full text stage. In this review, the pattern in suicide rates and suicide methods among elderly people between rural and urban areas has been described. However, it was not feasible to analyse the risk factors for suicide which remains an area warranting further investigation. The data analysed in this review were extracted from different included articles which derive their estimates from different sources and may be subject to differing biases. While we did assess risk of bias and consider this in the synthesis process [20], there remains nevertheless the possibility that some comparisons reflect methodological differences in study design or data sources, rather than genuine differences. It is possible that the same area in China might be classified in differing ways over time, due to the reason that China’s official definition of urban and rural areas based on the National Bureau of statistics of the People’s Republic of China has been updated over time. By necessity, we have relied on the use of this standard classification implemented by authors of included studies, but it is possible that some of the variation over time is due to changes in classification approaches.

### Implications for public health policy and practice

Although suicide rates have decreased dramatically in recent China, more attention should be paid to this preventable cause of death in both policy and health research. The rural–urban gap in elderly suicide rates is closing, but the suicide rate in rural areas remains nearly two times higher than the rate in urban areas. Moreover, it is commonly understood that restricting access to lethal suicide methods is one of the most effective suicide prevention strategies [53]. In rural areas where pesticides are commonly used in farming activities, the reduced accessibility of pesticides may have prevented some suicide attempts, and the reduced toxicity of pesticides in recent years may have led to increased resuscitation rates after these attempts. In recent years, and in response to the high rates of some suicide methods, many lethal methods such as pesticides have been banned for use in agriculture by law [36].

China has been witnessing considerable urbanisation over recent decades [39], with migration from rural areas to urban areas potentially resulting in a marked change to demographic characteristics which may have implications for suicide. For example, pesticide suicides in urban areas have increased in recent years while it used account for a lower proportion in urban areas [36]. Moreover, urbanisation may also trigger some mental health issues in specific populations (e.g. left-behind older adults, children or unemployed younger adults) and this can increase suicide risk [54]. Alongside the social changes and rapid economic growth in China, increased income and improved living standards with better health services, especially in rural areas, might have contributed to lower suicide rates [55].

## Conclusion and recommendation for future research

The suicide rate in China, especially in urban areas, is already one of the lowest in the world. But rates among elderly people have remained higher in rural than in urban areas. Therefore, suicide trends among elderly people in both rural and urban areas and their impact on overall suicide rates in China should be monitored. A notable feature of these figures is that elderly suicide rates among older adults in urban areas have slightly increased in recent years. Thus, attention should be paid to addressing suicide risk in elderly among urban, as well as rural, areas in suicide prevention strategies. Differences in the risk factors for suicide between regions should also be explored. Moreover, with the population in China experiencing rapid urbanisation and aging, it remains a challenge to ensure universal healthcare coverage which may mitigate suicide risk. Early articles also suggested that comparisons of suicide methods between different areas and cultures (e.g. different ethnic minorities) can help increase understanding of potential risk factors and provide valuable information for suicide prevention strategies [56]. For example, findings from early articles suggested that suicidal behaviour could linked with attitude towards suicide and it is important to identify factors that influence attitudes towards suicide in order to provide evidence for future prevention [57]. Our observation of important gender differences are also worthy of further exploration in future studies. The underlying reason why suicide rates between women and men are different can also be explored by analyse their social-economic status, culture background and other potential risk factors. In terms of future studies, the group of people who attempted suicide should be noticed since they could also benefit from the suicide prevention strategies. Furthermore, consideration should be given to the development of a suicide prevention strategy which includes a focus on the elderly, building on lessons from previous strategies implemented in China and elsewhere.

## Additional files

Additional file 1: Protocol of Systematic review. (DOCX 45 kb)

Additional file 2: Search strategy in PhycINFO (EBSCOhost), Medline (OVID) and EMBASE (OVID). (DOCX 18 kb)

Additional file 3: Reasons for exclusion of full-text articles. (DOCX 14 kb)

Additional file 4: Suicide rates in the included articles. (DOCX 21 kb)

Additional file 5: Results of risk of bias assessment of observational studies using the Newcastle-Ottawa Scale. (DOCX 19 kb)

Additional file 6: Numerical data extraction for meta-analysis. (DOCX 17 kb)

## Abbreviations

CNKI: China National Knowledge Infrastructure
ML: Meizhi Li
NOS: Newcastle-Ottawa Scale
OR(s): Odds Ratio(s)
SVK: Srinivasa Vittal Katikireddi
WHO: World Health Organization
