# Supplementary material for: Urban-rural inequalities in suicide among elderly people in China: a systematic review and meta-analysis
Source: Int J Equity Health. 2019 Jan 3;18:2. doi: 10.1186/s12939-018-0881-2 (PMC6319001; doi:10.1186/s12939-018-0881-2)
Supplement: Supplementary file 1 — Protocol of Systematic review. (DOCX 45 kb) [file 12939_2018_881_MOESM1_ESM.docx]

## Additional File 1. Protocol of Systematic review

**A systematic review of urban-rural inequalities in suicide in elderly people in China**

1. **Literature search**

Multiple terms in different concepts related to PECO (Population, Exposure, Context, Outcome) were used in both Chinese and English to systematically search for relevant articles.

Databases include EMBASE (Ovid), MEDLINE (Ovid), PsycINFO (EBSCOhost), CNKI (China National Knowledge Infrastructure, in Chinese) and PROSPREO (the International prospective register of systematic review). PROSPERO held by the University of York’s Centre for Reviews and Dissemination (1) and has been used to check what reviews are currently underway in the topic area.

Database: EMBASE (Ovid)

| Concept | Terms | Combined Hits (OR) |
| --- | --- | --- |
| **P**opulation | Elderly / Old people / Aged / Geriatric | 3,014,725 |
| **E**xposure | Rural / Urban / regional / geographic | 4,52,356 |
| **C**ontext | China / Chinese | 2,51,283 |
| **O**utcome | suicid* | 74,261 |
| Combine Hits (AND) |  | 139 |

Database: MEDLINE (Ovid)

| Concept | Terms | Combined Hits (OR) |
| --- | --- | --- |
| **P**opulation | Elderly / Old people / Aged / Geriatric | 2,345,933 |
| **E**xposure | Rural / Urban / regional / geographic | 474,130 |
| **C**ontext | China / Chinese | 324,162 |
| **O**utcome | suicid* | 74,120 |
| Combine Hits (AND) |  | 135 |

Database: PsychINFO (EBSCOhost)

| Concept | Terms | Combined Hits (OR) |
| --- | --- | --- |
| **P**opulation | Elderly / Geriatrics / older adults / elderly | 1,854,263 |
| **E**xposure | Rural / Urban / regional / geographic | 35,294 |
| **C**ontext | China / Chinese | 56,128 |
| **O**utcome | suicid* | 79,852 |
| **C**ombine Hits (AND) |  | 877 |

Database: CNKI (China National Knowledge Infrastructure, in Chinese)

| Concept | Terms | Combined Hits (OR) |
| --- | --- | --- |
| **P**opulation | 老年 | 479,239 |
| **E**xposure | 城乡 / 农村 / 城市 / 乡村 | 4,488,569 |
| **C**ontext [1] | ---- | ----- |
| **O**utcome | 自杀 | 49,663 |
| Combine Hits (AND) |  | 923 |

[1]: The context part was removed in the CNKI database to avoid the absence of related papers. This was because that articles published in the Chinese language sometimes did not highlight ‘China’ or ‘Chinese’ as its research regions in their research title, abstract or keywords.

Database: PROSPERO (the International prospective register of systematic review)

| Concept | Terms | Combined Hits (OR) |
| --- | --- | --- |
| **P**opulation | Elderly / Old people / Aged / Geriatric | 3 660 |
| **E**xposure | Rural / Urban / regional / geographic | 1 765 |
| **C**ontext | China / Chinese | 2 354 |
| **O**utcome | suicid* | 354 |
| Combine Hits (AND) |  | 0 |

1. **Background literature review**

The population in recent China has over 1.3 billion. China had experienced more than 250,000 suicides per year in the period between 1995 and 1999; suicide was the fifth-leading cause of death at that time (2). However, study in 2014 conducted by the Centre for Suicide Research and Prevention at the University of Hong Kong reported that China's suicide rate has decreased significantly, among the lowest levels in the world (3). In the meantime, a large rural-urban discrepancy and a high elderly-to-general-population suicide ratio have attracted attention (4). Later articles shows that the high rates in rural areas and among older adults are not that uncommon among Asian countries, for example, in South Korea, Japan, Taiwan and Hong Kong (5).

There are reasons to worry. From 2009 to 2011, 44% of all suicides occurred among those people aged 65 or above and 79% among rural residents (6). While suicide rates decrease dramatically in China, the rural elderly suicide rate remained very high and there were even some upward suicide trends among both urban and rural older adults in recent years (3, 7).

National studies on suicide indicate that suicidal behaviour and, in particular, the preferred suicide method, varies between regions. There also a large number of factors can affect the choice of suicide methods, for example, cultural concepts toward to suicide and the availability of those suicide methods (8). Regional comparisons of suicide methods help increase understanding of the interplay between rural and urban areas and provide basis information for preventive strategies (9).

It is necessary to explore how suicide method has affected historical suicide rates between rural and urban areas and to discuss their potential impact on suicide rates in the future. There are very few articles on the contributions of subgroups and method of suicide changes to trends in the total suicide rate. In this study, a systematic review was adopted, exploring region-specific, age-specific inequalities in suicide method. Analyse inequalities in rural-urban provides a detailed assessment on the suicide methods in China and will help to inform future suicide prevention policy, making it more focused and efficient.

1. **Aim**

This study aims to examine the temporal trends and differences in rural-urban suicide rates over recent decades since the suicide rate was available in China while taking into account a particular age group (elderly people) and methods of suicide.

1. **Research questions**
2. Is there a difference in model suicide rates between rural and urban areas among elderly people in China?
3. What are the differences in methods of suicide (in elderly people) between rural and urban areas in China?
4. Have trends over time in suicide rates differed between rural and urban areas in China?
5. **Intended research design**

Why systematic review?

Systematic review methodology will be used to locate and evaluate published literature on health inequality in suicide in elderly people between rural areas and urban areas in China. My previous search has found some relevant articles, and it is the most appropriate way to summarise the field before future research is conducted to look at the underlying determinants in rural-urban areas.

Why am I not doing primary research?

The primary research might be hampered by sample sizes and time constraints (1). Doing data collection to look at rural-urban suicide mode would be very time consuming, and is not feasible with the timescale.

**Methods**

**Databases:** Four main databases were checked, different searches were developed for different databases. However, same search strategies were used in these databases.

Electronic literature searches will be complete through EMBASE (Ovid), MEDLINE (Ovid), PsycINFO (EBSCOhost), CNKI (China National Knowledge Infrastructure, in Chinese). Papers in all languages were searched.

**Search strategies:**

Key search terms are identified to limit my searches and specify search parameters.

Subject headings or index terms are used to index the content of bibliographic databases. Reference lists and citation list were scanned manually.

Work with a medical librarian:

1. Suggestions about search terms;
2. Databases were recommended by medical librarian;
3. Set ending time for search;
4. Make a note for search maps each time;
5. Check search results after the final research finish.

While search strategy is applying, notes of results will be made. Screening of titles, index terms and available abstracts will also conduct among all search records.

**Inclusion and exclusion Criteria**

By searching different databases, full-text papers of all potentially eligible articles should be found. The relevance of each article will be assessed according to the inclusion criteria stated in the following table. Articles that do not meet the criteria will be excluded and their bibliographic details can be listed in an Appendix alongside reasons for their exclusion (1).

**Inclusion Criteria based on PECO**

**Type of studies:** All type of Studies that report suicide rate at all will be selected for this review.

**Population**: Elderly people

Explanation: Age >=60. In China, elderly people are defined on the basis of being over 60 years of age. Furthermore, the current retirement age for worker is 60.

**Exposure:** Rural and urban areas

Explanation: The standard of urban and rural areas(10): Rural and urban areas are defined by the National Bureau of Statistic of the People’s Republic of China, Based on the administrative division in China, According to actual construction, divided China into rural and urban areas. Actual construction refers to communal facilities, accommodations and other facilities are being built or already been built.

Any categorisation of geographical areas will be eligible, including national, province or city/town level area all acceptable.

**Context:** China

Explanation: Mainland China. Urban and rural areas are not defined consistently for areas outside of mainland China, therefore, Hong Kong, Macau and Taiwan will not be included.

**Outcome:** Primary: Suicide

Secondary: Suicide methods

Explanation: Only completed suicides are included in this systematic review. Moreover, because the secondary outcome of this project was suicide methods and one of the research questions was designed to explore the differences in suicide methods between elderly people in rural and urban areas in China, articles which considered the regional differences in elderly suicide methods will be included.

**Exclusion criteria**

Articles will be rejected if its abstract mismatches selection criteria.

Articles reserved in this step will be further evaluated with full text.

Furthermore, a review template will be developed to ensure standardised collection of the main information for each article.

**Data extraction approach**

For each eligible article, I will develop a template developed by the authors will use to extract key information from the article including features of selection bias, study design, and the prescribed outcomes. Data should be extracted strictly by use of the methods and instruments as instruments as stipulated to reduce the chance of observer bias. Relevant information is needed - the year of publication, year of data collection, first author for reference, name of the journal and category of publication (peer-reviewed paper or abstract), population size (denominator), number of deaths (numerator), cause, county, sample frame, etc.

Critical Appraisal Checklist: Newcastle-Ottawa

Included articles should be at low-risk bias and be critically.

Predefined inclusion criteria can minimize bias arising from the selective consideration of evidence and assess the risk of bias of included articles (11).

Selection bias might exist since only one reviewer in this review.

**Synthesis:**

This process brings the findings from the set of included articles together to draw conclusions based on the body of evidence. The two main approaches are quantitative (statistical pooling) and narrative synthesis (12).

The systematic review requires collection and analysis of all published data and bringing the data together. Data on suicide among elderly people between rural and urban areas during a period time could be found. In order to obtain clear and reliable results, we should decide whether it is possible to do meta-analysis and how can we combine the data. Two steps are thus important for the analysis: first, a complete collection of the published literature; second, the synthesis of the information acquired (13).

It is a better way to inspect the results in tables and graphs. A comprehensive table can help to bring the information together to answer the research questions.

1. **Hypothesis**

I hypothesise that the overall suicide rate in China has decreased, but regional disparity has increased. Also, suicide rates in elderly people are increasing while the overall suicide rate in national level is decreasing. There will be differences in suicide methods between rural and urban areas.

This study will help decision-makers to 1) Identify the current and potential problems regarding suicide among elderly people in rural and urban areas; 2) Understanding suicide methods in rural and urban areas will help guide suicide preventative actions (14).

1. **Ethical issues**

Reliable search engines were used. No harms will occur to individuals. However, findings from this research may include some geographic information. And how to share this resource properly is a question.

1. **Timetable**

| Action | Length of Time | End date |
| --- | --- | --- |
| Preliminary literature search | One month | Early January 2017 |
| Research topic and research questions | One month | Early February 2017 |
| Refining and check search strategy with medical librarian  Caring out searches | Run through the period of search stage | End of March 2017 |
| Developing and piloting data extraction template | Two weeks | Middle April 2017 |
| Data extraction | Two weeks | End of April 2017 |
| Critical Appraisal | Three weeks | Early May 2017 |
| Synthesis | Three weeks | End of May 2017 |
| Initial draft | One month | Early June 2017 |
| Final draft | One month | 7^th^ August 2017 |

1. **References:**

1. Boland A, Cherry MG, Dickson R. Doing  a systematic view: a student's guide2014.

2. Phillips MR, Li XY, Zhang YP. Suicide rates in China, 1995-99. Lancet. 2002;359(9309):835-40.

3. Wang CW, Chan CLW, Yip PSF. Suicide rates in China from 2002 to 2011: an update. Social Psychiatry and Psychiatric Epidemiology. 2014;49(6):929-41.

4. Sha F, Yip PSF, Law YW. Decomposing change in China's suicide rate, 1990-2010: ageing and urbanisation. Injury Prevention. 2017;23(1):40-5.

5. Chen YY, Wu KCC, Yousuf S, Yip PSF. Suicide in Asia: Opportunities and Challenges. Epidemiologic Reviews. 2012;34(1):129-44.

6. Li X, Xiao ZP, Xiao SF. Suicide among the elderly in mainland China. Psychogeriatrics. 2009;9(2):62-6.

7. Pritchard C, Baldwin DS. Elderly suicide rates in Asian and English-speaking countries. Acta Psychiatrica Scandinavica. 2002;105(4):271-5.

8. Farmer R, Rohde J. Effect of availability and acceptability of lethal instruments on suicide mortality - analysis of some International data. Acta Psychiatrica Scandinavica. 1980;62(5):436-46.

9. Nordentoft M, Qin P, Helweg-Larsen K, Juel K. Time-trends in method-specific suicide rates compared with the availability of specific compounds. The Danish experience. Nordic Journal of Psychiatry. 2006;60(2):97-106.

10. Statistical rules of the division of urban and rural in China. File No60. Beijing: The state Council; 2008.

11. Katikireddi SV, Egan M, Petticrew M. How do systematic reviews incorporate risk of bias assessments into the synthesis of evidence? A methodological study. Journal of Epidemiology and Community Health. 2015;69(2):189-95.

12. Popay J, Roberts H, Sowden A, Petticrew M, Arai L, Rodgers M, et al. Guidance on the Conduct of Narrative Synthesis in Systematic Reviews. A Product from the ESRC Methods Programme2006.

13. Chappell F. Meta-analysis in medical research: The handbook for the understanding and practice of meta-analysis. Journal of the Royal Statistical Society Series a-Statistics in Society. 2005;168:875-6.

14. Huang YH, Wu QJ, Li LL, Li D, Li J, Zhou C, et al. Different extent in decline of infant mortality by region and cause in Shenyang, China. Scientific Reports. 2016;6.
