# Supplementary material for: Urban-rural inequalities in suicide among elderly people in China: a systematic review and meta-analysis
Source: Int J Equity Health. 2019 Jan 3;18:2. doi: 10.1186/s12939-018-0881-2 (PMC6319001; doi:10.1186/s12939-018-0881-2)
Supplement: Supplementary file 2 — Search strategy in PhycINFO (EBSCOhost), Medline (OVID) and EMBASE (OVID). (DOCX 18 kb) [file 12939_2018_881_MOESM2_ESM.docx]

## Additional File 2. Search strategy in PhycINFO (EBSCOhost), Medline (OVID) and EMBASE (OVID)

**PhycINFO (EBSCOhost) Search History**

| **#** | **Query** | **Results** |
| --- | --- | --- |
| S12 | S5 AND S8 AND S11 | 981 |
| S11 | S9 OR S10 | 80,023 |
| S10 | TI suicid* OR AB suicid* | 53,273 |
| S9 | DE "Suicide" OR DE "Death and Dying" OR DE "Suicidal Ideation" OR DE "Suicide Prevention" OR DE "Suicidology" | 59,180 |
| S8 | S6 OR S7 | 58,910 |
| S7 | TI ( China or Chinese ) OR AB ( China or Chinese ) OR PL ( China or Chinese ) | 58,685 |
| S6 | DE "Chinese Cultural Groups" | 5,386 |
| S5 | S1 OR S2 OR S3 OR S4 | 1,857,253 |
| S4 | TI ( (age or years or over) N5 (60 or 50 or 70 or 80 or 85) ) OR AB ( (age or years or over) N5 (60 or 50 or 70 or 80 or 85) ) OR AG ( (age or years or over) N5 (60 or 50 or 70 or 80 or 85) ) | 58,287 |
| S3 | TI ( elder* or aged or centenarian or nonagenarian* or octogenarian* or old* or geriatric* ) OR AB ( elder* or aged or centenarian or nonagenarian* or octogenarian* or old* or geriatric* ) OR AG ( elder* or aged or centenarian or nonagenarian* or octogenarian* or old* or geriatric* ) | 1,834,394 |
| S2 | DE "Geropsychology" OR DE "Geriatrics" | 10,838 |
| S1 | DE "Aging" OR DE "Gerontology" | 70,415 |

**MEDLINE/EMBASE (Ovid)**

Search terms

1. exp Aged/

2. Geriatrics/

3. (elder* or aged or centenarian or nonagenarian* or octogenarian* or old* or gerontolog* or geriatric*).tw.

4. ((age or years or over) adj5 ("60" or "65" or "70" or "80" or "85")).tw.

5. 1 or 2 or 3 or 4

6. exp China/

7. (China or Chinese).tw.

8. 6 or 7

9. exp Suicide/

10. suicid*.tw.

11. 9 or 10

12. 5 and 8 and 11
