# Supplementary material for: Urban-rural inequalities in suicide among elderly people in China: a systematic review and meta-analysis
Source: Int J Equity Health. 2019 Jan 3;18:2. doi: 10.1186/s12939-018-0881-2 (PMC6319001; doi:10.1186/s12939-018-0881-2)
Supplement: Supplementary file 3 — Reasons for exclusion of full-text articles. (DOCX 14 kb) [file 12939_2018_881_MOESM3_ESM.docx]

**Additional File 3. Reasons for exclusion of full-text articles**

| **Reasons for exclusion** | **Number** |
| --- | --- |
| **Population** | |
| Population age group unsuitable | 9 |
| **Exposure** | |
| Did not report elderly rural and urban data or differences between these areas | 17 |
| **Context** | |
| Not about Mainland China | 7 |
| **Outcome** | |
| Not completed suicide(e.g. Attempted suicide, self-harm, injury, mortality, death) | 23 |
| Reasons or risk factors for suicide only | 17 |
| **Article unobtainable** | 4 |
| **Total number** | 77 |
