# Supplementary material for: Urban-rural inequalities in suicide among elderly people in China: a systematic review and meta-analysis
Source: Int J Equity Health. 2019 Jan 3;18:2. doi: 10.1186/s12939-018-0881-2 (PMC6319001; doi:10.1186/s12939-018-0881-2)
Supplement: Supplementary file 4 — Suicide rates in the included articles. (DOCX 21 kb) [file 12939_2018_881_MOESM4_ESM.docx]

**Additional File 4. Suicide rates in the included articles**

| **Author** | **Year** | **Region** | **Study period** | **Age group** | **Suicide rate (Per 100,000)** | | | | | | | |
| --- | --- | --- | --- | --- | --- | --- | --- | --- | --- | --- | --- | --- |
|  |  |  |  |  | **Rural** | **Urban** |  | **Rural women** | **Urban women** |  | **Rural men** | **Urban men** |
| Ji, J., et al | 2001 | National | 1988 | All | 27.49 | 9.02 |  | 30.40 | 10.53 |  | 23.08 | 7.99 |
|  |  |  | 1990 | All | 22.46 | 8.57 |  | 24.64 | 9.07 |  | 20.35 | 8.10 |
|  |  |  | 1992 | All | 25.43 | 8.46 |  | 27.07 | 9.29 |  | 23.18 | 7.69 |
| Lu, J., et al | 2013 | YunNan province | 2004-2005 | 60-84 | 65.37 | 65.96 |  | 62.99 | 67.99 |  | 48.90 | 67.99 |
|  |  |  |  | All | 20.60 | 19.18 |  | 18.93 | 18.05 |  | 24.32 | 20.19 |
| Phillips, M. et al | 2002 | National | 1995-1999 | 60-84 | 82.80 | 16.70 |  | 77.90 | 16.10 |  | 88.00 | 17.30 |
|  |  |  |  | All | 27.10 | 8.30 |  | 30.50 | 8.30 |  | 23.90 | 8.30 |
| Sun, J., et al | 2013 | Shandong  Province | 1991-1995 | All | 40.02 | 6.62 |  | 40.75 | 6.88 |  | 39.26 | 6.58 |
|  |  |  | 1996-2000 | All | 31.16 | 7.71 |  | 30.28 | 6.87 |  | 32.04 | 8.82 |
|  |  |  | 2001-2005 | All | 21.24 | 4.95 |  | 18.99 | 3.97 |  | 23.55 | 5.87 |
|  |  |  | 2006-2010 | All | 19.00 | 5.32 |  | 17.33 | 4.73 |  | 20.56 | 5.90 |
| Sun, L., et al | 2014 | National | 1987-1994 | 65-74 |  |  |  | 67.12 | 13.98 |  | 88.12 | 25.70 |
|  |  |  |  | 75+ |  |  |  | 93.85 | 45.75 |  | 134.20 | 51.75 |
|  |  |  | 2007 | 65-69 |  |  |  | 27.35 | 16.66 |  | 36.35 | 21.26 |
|  |  |  |  | 70-74 |  |  |  | 36.25 | 24.27 |  | 52.98 | 33.72 |
|  |  |  |  | 75-79 |  |  |  | 46.28 | 34.34 |  | 72.09 | 44.84 |
| Yip, P. S., et al | 2000 | Beijing city | 1991-1996 | 65-74 |  |  |  | 29.70 | 12.60 |  | 45.00 | 15.40 |
|  |  |  |  | 75+ |  |  |  | 55.10 | 22.90 |  | 98.80 | 36.80 |
| Yip, P. S. F. | 2001 | National | 1987-1996 | 65-74 |  |  |  | 28.90 | 12.30 |  | 46.40 | 16.50 |
|  |  |  |  | 75+ |  |  |  | 64.20 | 26.60 |  | 109.10 | 38.30 |
| Zhong, B. L., et al (b) | 2016 | National | 2013-2014 | 65+ | 39.68 | 19.94 |  |  |  |  |  |  |
|  |  |  |  | 65-74 | 29.00 | 15.49 |  |  |  |  |  |  |
|  |  |  |  | 75+ | 57.58 | 27.38 |  |  |  |  |  |  |
| Peng.Z., et al | 2013 | Chengdu city | 2006-2010 | 60-69 | 12.74 | 3.81 |  |  |  |  |  |  |
|  |  |  |  | 70-79 | 22.10 | 8.34 |  |  |  |  |  |  |
|  |  |  |  | 80+ | 35.36 | 10.76 |  |  |  |  |  |  |
| Xu.Z.G., et al | 1993 | Yantai city and Laiyang | 1988-1990 | 60+ |  |  |  | 68.90 | 25.20 |  | 85.76 | 25.63 |
| Xu.H.L., et al | 2000 | Hunan Province | 1990-1998 | 60+ | 88.30 | 22.10 |  | 61.90 | 21.70 |  | 107.60 | 22.40 |
| Li.D.Y., et al | 2007 | Zhuhai city | 2004-2005 | 60-69 | 50.51 | 13.69 |  |  |  |  |  |  |
|  |  |  |  | 70-79 | 97.61 | 12.57 |  |  |  |  |  |  |
|  |  |  |  | 80+ | 190.79 | 31.04 |  |  |  |  |  |  |
| Cai.Y., et al | 2012 | National | 2010 | 60+ | 50.76 | 19.60 |  |  |  |  |  |  |
| Wang, W.L | 2013 | National | 2010 | 60-64 | 19.80 | 7.50 |  |  |  |  |  |  |
|  |  |  |  | 65-69 | 23.76 | 9.75 |  |  |  |  |  |  |
|  |  |  |  | 70-74 | 35.64 | 13.50 |  |  |  |  |  |  |
|  |  |  |  | 75-79 | 53.46 | 20.25 |  |  |  |  |  |  |
|  |  |  |  | 80-84 | 81.18 | 29.25 |  |  |  |  |  |  |
|  |  |  |  | 85+ | 104.94 | 30.75 |  |  |  |  |  |  |
| Yan, T.J | 2003 | National | 1990 | 60+ | 65.42 | 24.90 |  |  |  |  |  |  |
|  |  |  | 1995 | 60+ | 76.24 | 19.98 |  |  |  |  |  |  |
|  |  |  | 2000 | 60+ | 74.73 | 15.56 |  |  |  |  |  |  |
