# Supplementary material for: Urban-rural inequalities in suicide among elderly people in China: a systematic review and meta-analysis
Source: Int J Equity Health. 2019 Jan 3;18:2. doi: 10.1186/s12939-018-0881-2 (PMC6319001; doi:10.1186/s12939-018-0881-2)
Supplement: Supplementary file 5 — Results of risk of bias assessment of observational studies using the Newcastle-Ottawa Scale. (DOCX 19 kb) [file 12939_2018_881_MOESM5_ESM.docx]

**Additional File 5. Results of risk of bias assessment of observational studies using the Newcastle-Ottawa Scale**^^[[1]](#footnote-1)^^

| **Author** | **Year** | **Selection (Worth four points)** | | | | **Comparability (Worth two points)** | **Outcome (Worth three points)** | | | **Score** |
| --- | --- | --- | --- | --- | --- | --- | --- | --- | --- | --- |
|  |  | **Representativeness of the sample population (One point)** | **Consistency of population in rural and urban group (One point)** | **Study period clear (One point)** | **Source of Data (One point)** | **Study controls for other variables (Two point)** | **Assessment of the outcome (One point)** | **Presentation of the results (One point)** | **Exploring the trends over time (One point)** |  |
| He, Z. X., et al | 1998 | 1 | 0 | 1 | 0 | 0 | 1 | 0 | 1 | 4 |
| Ji, J., et al | 2001 | 1 | 1 | 1 | 1 | 1 | 1 | 0 | 1 | 7 |
| Li, X., et al | 2009 | 1 | 0 | 0 | 0 | 0 | 1 | 1 | 0 | 3 |
| Lu, J., et al | 2013 | 0 | 1 | 1 | 1 | 1 | 1 | 0 | 0 | 5 |
| Page, A., et al | 2017 | 1 | 1 | 1 | 1 | 2 | 1 | 0 | 1 | 8 |
| Phillips, M., et al | 2002 | 1 | 1 | 1 | 1 | 2 | 1 | 1 | 0 | 8 |
| Sha, F., et al | 2017 | 1 | 1 | 1 | 1 | 1 | 0 | 0 | 1 | 6 |
| Sun, J., et al | 2013 | 0 | 1 | 1 | 1 | 1 | 1 | 0 | 1 | 6 |
| Sun, L., et al | 2014 | 1 | 1 | 1 | 1 | 1 | 0 | 1 | 1 | 7 |
| Wang, C. W., et al | 2014 | 1 | 1 | 1 | 1 | 2 | 1 | 1 | 1 | 9 |
| Yang, G. H., et al | 2005 | 1 | 1 | 1 | 1 | 0 | 0 | 1 | 1 | 6 |
| Yip, P. S., et al | 2000 | 0 | 1 | 1 | 1 | 1 | 1 | 0 | 1 | 6 |
| Yip, P. S., et al | 2005 | 1 | 1 | 1 | 1 | 1 | 0 | 1 | 1 | 6 |
| Yip, P. S., et al | 2008 | 1 | 1 | 1 | 1 | 1 | 0 | 1 | 1 | 7 |
| Yip, P. S. F. | 2001 | 0 | 1 | 1 | 1 | 1 | 0 | 1 | 0 | 8 |
| Zhong, B. L., et al (a) | 2016 | 1 | 1 | 1 | 1 | 2 | 1 | 1 | 1 | 9 |
| Zhong, B. L., et al (b) | 2016 | 1 | 1 | 1 | 1 | 2 | 1 | 1 | 0 | 8 |
| Peng, Z., et al (In Chinese) | 2013 | 0 | 1 | 1 | 1 | 1 | 1 | 1 | 0 | 6 |
| Xu Z. G., et al (in Chinese) | 1993 | 0 | 1 | 1 | 1 | 0 | 1 | 1 | 0 | 5 |
| Xu, H. L., et al (in Chinese) | 2000 | 0 | 1 | 1 | 1 | 2 | 1 | 1 | 1 | 8 |
| Li D. Y., et al (in Chinese) | 2007 | 0 | 1 | 1 | 1 | 2 | 1 | 1 | 0 | 5 |
| Cai, Y., et al (in Chinese) | 2012 | 1 | 1 | 1 | 1 | 1 | 1 | 1 | 1 | 8 |
| Wang W.L (in Chinese) | 2013 | 1 | 0 | 1 | 1 | 1 | 1 | 1 | 1 | 7 |
| Yan T.J (in Chinese) | 2003 | 1 | 1 | 1 | 1 | 2 | 1 | 1 | 1 | 9 |

1. The lowest possible score for this scale is zero and the highest possible score is nine. Each item is worth highest one point (except comparability part worth two points) and lowest zero point. [↑](#footnote-ref-1)
