# Supplementary material for: Urban-rural inequalities in suicide among elderly people in China: a systematic review and meta-analysis
Source: Int J Equity Health. 2019 Jan 3;18:2. doi: 10.1186/s12939-018-0881-2 (PMC6319001; doi:10.1186/s12939-018-0881-2)
Supplement: Supplementary file 6 — Numerical data extraction for meta-analysis. (DOCX 17 kb) [file 12939_2018_881_MOESM6_ESM.docx]

**Additional File 6. Numerical data extraction for meta-analysis**

| **Author** | **Year of Publication** | **Age group** | **Population** | **Rural** | | | **Urban** | | |
| --- | --- | --- | --- | --- | --- | --- | --- | --- | --- |
|  |  |  |  | **Number of total population** | **Number of total suicide case** | **Total rate (1/100,000)** | **Number of total population** | **Number of total suicide case** | **Total rate (1/100,000)** |
| Lu, J., et al | 2013 | 60-84 | Total | 336549 |  | 65.37 | 386682 |  | 65.96 |
|  |  |  | Women | 176230 |  | 62.99 | 200410 |  | 48.90 |
|  |  |  | Men | 160322 |  | 67.99 | 186277 |  | 67.99 |
| Phillips, M. et al | 2002 | 60-84 | Total | 95000000 |  | 82.80 | 27300000 |  | 16.70 |
|  |  |  | Women | 49200000 |  | 77.90 | 13900000 |  | 16.10 |
|  |  |  | Men | 45800000 |  | 88.00 | 13400000 |  | 17.30 |
| Zhong, B. L., et al (b) | 2016 | 65+ | Total | 87910000 |  | 39.68 | 31020000 |  | 19.94 |
|  |  | 65-74 | Total | 54800000 |  | 29.00 | 19300000 |  | 15.49 |
|  |  | 75+ | Total | 33120000 |  | 57.58 | 11720000 |  | 27.38 |
| Peng.Z., et al | 2013 | 60-69 | Total |  | 342 | 12.74 |  | 40 | 3.81 |
|  |  | 70-79 | Total |  | 335 | 22.10 |  | 44 | 8.34 |
|  |  | 80+ | Total |  | 171 | 35.36 |  | 18 | 10.76 |
| Xu.Z.G., et al | 1993 | 60+ | Total |  | 196 |  |  | 22 |  |
|  |  |  | Female |  | 97 | 68.09 |  | 12 | 25.20 |
|  |  |  | Male |  | 99 | 85.76 |  | 10 | 25.63 |
| Li.D.Y, et al | 2007 | 60-69 | Total |  | 19 | 50.51 |  | 5 | 13.69 |
|  |  | 70-79 | Total |  | 24 | 97.61 |  | 3 | 12.57 |
|  |  | 80+ | Total |  | 19 | 190.79 |  | 3 | 31.04 |
| Cai.Y., et al | 2012 | 60+ | Total |  | 2965 | 50.76 |  | 825 | 19.60 |
